# Life Expectancy in Survivors of Esophageal Cancer Compared with the Background Population

**DOI:** 10.1245/s10434-022-11416-4

**Published:** 2022-02-21

**Authors:** Ellinor Lundberg, Pernilla Lagergren, Fredrik Mattsson, Jesper Lagergren

**Affiliations:** 1 Upper Gastrointestinal Surgery, Department of Molecular Medicine and Surgery, Karolinska Institutet, Karolinska University Hospital, Stockholm, Sweden; 2 Surgical Care Science, Department of Molecular Medicine and Surgery, Karolinska Institutet, Karolinska University Hospital, Stockholm, Sweden; 3 Department of Surgery and Cancer, Imperial College London, London, UK; 4 School of Cancer and Pharmaceutical Sciences, King’s College London, London, UK

## Abstract

It is unknown whether the survival of patients cured of esophageal cancer differs from that of the corresponding background population. This nationwide and population-based cohort study included all patients who survived for at least 5 years after surgery for esophageal cancer in Sweden between 1987 and 2015, with follow-up throughout 2020. Relative survival rates with 95% confidence intervals (95% CI) were calculated by dividing the observed with the expected survival. The expected survival was assessed from the entire Swedish population of the corresponding age, sex, and calendar year. Yearly relative survival rates were calculated between 6 and 10 years postoperatively. Among all 762 participants, the relative survival was initially similar to the background population (96.1%, 95% CI 94.3–97.9%), but decreased each following postoperative year to 83.5% (95% CI 79.5–87.6%) by year 10. The drop in relative survival between 6 and 10 years was more pronounced in participants with a history of squamous cell carcinoma [from 94.5% (95% CI 91.2–97.8%) to 70.8% (95% CI 64.0–77.6%)] than in those with adenocarcinoma [from 96.9% (95% CI 94.8–99.0%) to 91.5% (95% CI 86.6–96.3%)], and in men [from 96.0% (95% CI 93.8–98.1%) to 81.8% (95% CI 76.8–86.8%)] than in women [from 96.4% (95% CI 93.4–99.5%) to 88.1% (95% CI 81.5–94.8%)]. No major differences were found between age groups. In conclusion, esophageal cancer survivors had a decline in survival between 6 and 10 years after surgery compared with the corresponding general population, particularly those with a history of squamous cell carcinoma of the esophagus and male sex.

Esophageal cancer is the eighth most common cancer worldwide, with approximately 600,000 new cases every year,^1,2^ and has a poor prognosis with overall 5-year survival below 20%.^2^ The dominating histological types are adenocarcinoma, which is mainly associated with gastroesophageal reflux disease and obesity, and squamous cell carcinoma, with heavy alcohol consumption and tobacco smoking as the main risk factors.^2–4^ Curatively intended treatment usually includes surgical resection (esophagectomy) with or without pre- or perioperative chemo(radio)therapy and is followed by a 5-year survival of 30–45%, with tumor stage as the strongest prognostic factor.^2,6,7^ Esophagectomy is one of the most extensive standard surgical procedures, and comorbidity and fitness are carefully assessed preoperatively. Tumor recurrences and deaths in esophageal cancer typically occur shortly after treatment (within 1–3 years), and 5-year survivors can be considered cured.^1^ Whether life expectancy among esophageal cancer survivors differs from the corresponding background population is unknown. Yet this knowledge is of great relevance to individuals and families, and also to healthcare and society. Esophageal cancer survivors might have a worse survival; the risk factors for the tumor could increase the risk of other lethal diseases or conditions, the treatment itself can cause serious sequelae,^7,8^ and the prevalence of severe comorbidities could be increased.^7,9,10^ The survival may also be better, e.g., due to selection of the fittest patients for curative treatment, healthy lifestyle changes, and increased awareness of symptoms of serious illnesses that may alert individuals to seek medical attention in time. This study aimed to clarify whether and how the survival of patients cured of esophageal cancer differs from that of the corresponding general population.

## Patients and Methods

### Design

This was a population-based cohort study that included all individuals in Sweden who survived for at least 5 years after surgery for esophageal cancer within the study period from 1 January 1987 to 31 December 2020. The observed survival from 6 to 10 years after esophagectomy in this cohort was compared with the expected survival, which was estimated from the survival in the entire Swedish population of the corresponding age, sex, and calendar year. We did not assess longer follow-up after considering the statistical power. The dominating surgical procedure was open thoracoabdominal surgery according to Ivor Lewis. A patient research partnership group consisting of former esophageal cancer patients was involved in formulating the research question and in the planning of the study. The study was approved by the Regional Ethical Review Board in Stockholm, Sweden.

### Data Sources

#### Study Cohort

The cohort of esophageal cancer survivors was retrieved from the Swedish Esophageal Cancer Surgery Study (SESS). Earlier versions of this cohort have been used for studies examining factors influencing the survival after esophageal cancer surgery.^11–13^ Data came from medical records and national Swedish health data registries, including the Cancer Registry, Patient Registry, and Cause of Death Registry. The Cancer Registry is 98% complete for esophageal cancer;^14^ the Patient Registry has a positive predictive value of 99.6% for esophageal cancer surgery;^15^ the recording of death dates is 100% in the Cause of Death Registry.^16^

#### Background Population

Statistics Sweden provided survival data of the entire Swedish population by age, sex, and calendar year. Statistics Sweden also records the death dates of all Swedish residents, and this information is automatically issued to the Cause of Death Registry.^17^ The completeness of the recording of residents and deaths (including deaths abroad) is 100%.^16^ Ethnicity data was not available for the cohort nor the background population.

### Statistical Analysis

The follow-up of the esophageal cancer survivors started in year 6 after esophagectomy and ended after year 10. Relative survival rates with 95% confidence intervals (CI) were calculated for each of the five follow-up years by dividing the observed survival with the expected survival using the life table method.^18^ The expected survival was derived from the entire Swedish population of the same age, sex, and calendar year. Stratified analyses were performed by sex (male and female), age (in quartiles, i.e., four approximately equal-sized groups), calendar period (quartiles), tumor histology (adenocarcinoma and squamous cell carcinoma), and comorbidity (Charlson comorbidity index 0, 1, and ≥ 2). Calendar year and age groups were divided into quartiles to avoid arbitrary cut-offs as decided before initiating the analyses. A sub-analysis was performed in which we excluded patients with diagnoses related to heavy smoking tobacco and alcohol abuse, in both patients with a history of adenocarcinoma and those with squamous cell carcinoma. The analyses followed a predefined study protocol and were conducted by an experienced biostatistician (F.M.) who used the statistical software SAS, Version 9.4 (SAS Institute Inc., Cary, NC, USA).

## Results

### Participants

The study included 762 individuals who survived for at least 5 years after surgery for esophageal cancer. The majority of the participants were men, aged above 70 years, who underwent their surgery after the year 2007, and had tumor histology of adenocarcinoma. The vast majority of the patients had early pathological tumor stage (Table 1).

**Table 1:** Survivors of esophageal cancer in Sweden from 1987–2020

	Number** (%)**
Total	762 (100.0)
*Sex*	
Men	552 (72.4)
Women	210 (27.6)
*Age (in years)*	
< 62	166 (21.8)
62–69	211 (27.7)
70–73	117 (15.4)
> 73	268 (35.2)
*Calendar period*	
1992–1999	72 (9.4)
2000–2007	171 (22.4)
2008–2015	214 (28.1)
2016–2020	305 (40.0)
*Tumor histology*	
Adenocarcinoma	493 (64.7)
Squamous cell carcinoma	264 (34.6)
*Pathological tumor stage*	
0–I	407 (53.4)
II	207 (27.2)
III	120 (15.7)
IV	20 (2.6)
Missing	8 (1.0)
*Resection margins*	
R0	656 (86.0)
R1 or R2	44 (5.8)
Missing	62 (8.1)

### Survival

#### Total Cohort

The survival of the total cohort was marginally lower than the background population 6 years after surgery (96.1%, 95% CI 94.3–97.9), but decreased almost linearly each year to 83.5% (95% CI 79.5–87.6) in year 10 (Table 2).

**Table 2:** Survival (percentage and 95% confidence intervals) between 6 and 10 years after surgery for esophageal cancer, all participants

Total			
Year	Number	Observed survival	Relative survival
6	762	93.7 (92.0–95.4)	96.1 (94.3–97.9)
7	653	87.7 (85.3–90.1)	92.1 (89.6–94.6)
8	540	82.8 (80.0–85.6)	89.4 (86.4–92.4)
9	463	78.3 (75.2–81.5)	86.9 (83.4–90.4)
10	391	73.1 (69.6–76.7)	83.5 (79.5–87.6)

#### Sexes

The reduction in relative survival during the follow-up decreased more in men [from 96.0% (95% CI 93.8–98.1%) in year 6 to 81.8% (95% CI 76.8-86.8%) in year 10] than in women [from 96.4% (95% CI 93.4–99.5%) in year 6 to 88.1% (95% CI 81.5–94.8%) in year 10] (Table 3).

**Table 3:** Survival (percentage and 95% confidence intervals) between 6 and 10 years after surgery for esophageal cancer, stratified by sex

Year	Number	Observed survival	Relative survival
*Men*			
6	552	93.3 (91.2–95.4)	96.0 (93.8–98.1)
7	466	86.9 (84.0–89.8)	91.8 (88.7–94.8)
8	388	82.0 (78.6–85.4)	89.2 (85.5–92.9)
9	334	76.8 (73.0–80.6)	86.1 (81.8–90.4)
10	274	70.6 (66.4–74.9)	81.8 (76.8–86.8)
*Women*			
6	210	94.8 (91.8–97.8)	96.4 (93.4–99.5)
7	187	89.7 (85.5–93.9)	93.0 (88.6–97.3)
8	152	85.0 (79.9–90.1)	89.9 (84.5–95.3)
9	129	82.3 (76.8–87.9)	89.0 (83.0–95.0)
10	117	79.5 (73.5–85.5)	88.1 (81.5–94.8)

#### Age Groups

There were no major differences in the decline in relative survival when comparing the different age groups (Table 4).

**Table 4:** Survival (percentage and 95% confidence intervals) between 6 and 10 years after surgery for esophageal cancer, stratified by age grou*p*

Year	Number	Observed survival	Relative survival
*Age < 62 years*			
6	166	97.0 (94.4–99.6)	97.4 (94.8–100)
7	152	94.4 (90.9–98.0)	95.3 (91.7–98.9)
8	134	89.5 (84.6–94.4)	90.8 (85.9–95.8)
9	117	85.7 (80.0–91.4)	87.5 (81.6–93.3)
10	103	83.2 (77.0–89.4)	85.4 (79.0–91.8)
*Age 62–69 years*			
6	211	94.8 (91.8–97.8)	95.9 (92.8–98.9)
7	180	90.1 (85.9–94.2)	92.2 (88.0–96.5)
8	152	87.1 (82.3–91.8)	90.4 (85.5–95.4)
9	136	82.6 (77.1–88.2)	87.1 (81.2–92.9)
10	117	77.0 (70.6–83.4)	82.4 (75.6–89.3)
*Age 70–73 years*			
6	117	96.6 (93.3–99.9)	98.5 (95.1–101.9)
7	104	92.9 (88.1–97.6)	96.8 (91.8–101.8)
8	86	83.2 (75.8–90.5)	88.8 (80.9–96.7)
9	71	78.5 (70.2–86.7)	86.1 (77.0–95.2)
10	56	72.9 (63.5–82.2)	82.1 (71.6–92.6)
*Age > 73 years*			
6	268	89.6 (85.9–93.2)	94.3 (90.5–98.2)
7	217	79.2 (74.2–84.2))	88.0 (82.4–93.5)
8	168	75.0 (69.6–80.4	89.2 (82.7–95.7)
9	139	70.1 (64.2–76.1)	89.4 (81.8–97.0)
10	115	63.4 (56.9–70.0)	87.8 (78.7–96.9)

#### Calendar Periods

The relative survival showed similar trends, with a reduction between 6 and 10 years after esophagectomy in all calendar periods, but the survival was worse during earlier calendar periods than in more recent periods (Table 5).

**TABLE 5:** Survival (percentage and 95% confidence intervals) between 6 and 10 years after surgery for esophageal cancer, stratified by calendar period

Year	Number	Observed survival	Relative survival
*Year 1992–1999*			
6	72	86.1 (78.1–94.1)	89.2 (80.9–97.5)
7	62	75.0 (65.0–85.0)	80.1 (69.4–90.8)
8	54	68.1 (57.3–78.8)	74.6 (62.8–86.4)
9	49	59.7 (48.4–71.1)	67.8 (54.9–80.6)
10	43	52.8 (41.3–64.3)	62.1 (48.5–75.7)
*Year 2000–2007*			
6	171	90.6 (86.3–95.0)	93.2 (88.7–97.7)
7	155	85.4 (80.1–90.7)	90.1 (84.5–95.7)
8	146	81.9 (76.1–87.7)	88.8 (82.5–95.1)
9	140	78.4 (72.2–84.5)	87.3 (80.4–94.2)
10	134	73.1 (66.5–79.8)	83.8 (76.2–91.5)
*Year 2008–2015*			
6	214	95.8 (93.1–98.5)	97.7 (95.0–100.5)
7	205	90.7 (86.8–94.6)	94.5 (90.5–98.6)
8	194	85.1 (80.3–89.8)	90.7 (85.6–95.8)
9	182	80.8 (75.6–86.1)	88.4 (82.6–94.2)
10	173	76.6 (71.0–82.3)	86.0 (79.6–92.3)
*Year 2016–2020*			
6	305	95.7 (93.5–98.0)	98.1 (95.8–100.5)
7	231	89.9 (86.3–93.6)	94.4 (90.6–98.3)
8	146	85.6 (81.0–90.3)	93.1 (88.0–98.1)
9	92	81.9 (76.2–87.6)	91.8 (85.4–98.2)
10	41	75.9 (67.5–84.3)	89.7 (79.7–99.6)

#### Tumor Histology

There was a pronounced drop in relative survival in participants with a history of squamous cell carcinoma, i.e., from 94.5% (95% CI 91.2–97.8%) in year 6 to 70.8% (95% CI 64.0–77.6%) in year 10. Participants with a history of adenocarcinoma indicated only a slightly decreased relative survival during follow-up, with a reduction from 96.9% (95% CI 94.8–99.0%) in year 6 to 91.5% (95% CI 86.6–96.3%) in year 10 (Table 6).

**Table 6:** Survival (percentage and 95% confidence intervals) between 6 and 10 years after surgery for esophageal cancer, stratified by tumor histology

Year	Number	Observed survival	Relative survival
*Adenocarcinoma*			
6	493	94.3 (92.3–96.4)	96.9 (94.8–99.0)
7	416	89.6 (86.8–92.3)	94.4 (91.5–97.3)
8	336	86.1 (82.9–89.3)	93.7 (90.2–97.3)
9	286	84.3 (80.8–87.8)	94.6 (90.7–98.5)
10	243	78.7 (74.6–82.9)	91.5 (86.6–96.3)
*Squamous cell carcinoma*			
6	264	92.4 (89.2–95.6)	94.5 (91.2–97.8)
7	232	84.1 (79.6–88.5)	87.8 (83.1–92.4)
8	201	77.0 (71.7–82.2)	81.9 (76.4–87.5)
9	174	68.6 (62.7–74.4)	74.6 (68.2–80.9)
10	145	63.8 (57.7–70.0)	70.8 (64.0–77.6)

#### Comorbidity

The relative survival of esophageal cancer survivors was worse in patients with higher Charlson comorbidity scores (Table 7).

**Table 7:** Survival (percentage and 95% confidence intervals) between 6 and 10 years after surgery for esophageal cancer, stratified by Charlson comorbidity index

Year	Number	Observed survival	Relative survival
*Charlson comorbidity index 0*			
6	355	95.8 (93.7–97.9)	98.0 (95.9–100.1)
7	312	91.2 (88.2–94.2)	95.5 (93.3–98.6)
8	258	87.6 (84.0–91.3)	93.9 (90.1–97.8)
9	225	83.7 (79.6–87.9)	91.5 (86.9–96.1)
10	191	79.8 (75.1–84.5)	89.3 (84.0–94.6)
*Charlson comorbidity index 1*			
6	250	94.4 (91.6–97.3)	96.7 (93.8–99.6)
7	217	85.7 (81.2–90.2)	89.9 (85.2–94.6)
8	178	74.6 (68.7–80.4)	86.2 (80.5–91.8)
9	149	74.6 (68.7–80.4)	83.0 (76.5–87.5)
10	121	68.4 (61.9–74.9)	78.4 (71.0–85.9)
*Charlson comorbidity index 2*			
6	157	87.9 (82.8–93.0)	90.7 (85.5–96.0)
7	124	82.9 (76.9–88.9)	88.1 (81.7–94.4)
8	104	76.6 (69.6–83.5)	84.4 (76.7–92.1)
9	89	72.3 (64.7–79.8)	82.9 (74.3–91.5)
10	79	65.9 (57.6–74.1)	78.7 (68.9–88.6)

#### Exclusion of Patients with Smoking- and Alcohol-Related Diagnoses

The relative survival remained inferior to the corresponding background population after exclusion of patients with smoking- and alcohol-related diagnoses. There were minor improvements in relative survival in patients with a history of adenocarcinoma and a moderate improvement in those with a history of squamous cell carcinoma (Table 8).

**Table 8:** Survival (percentage and 95% confidence intervals) between 6 and 10 years after surgery for esophageal cancer, excluding patients with diagnoses related to heavy smoking tobacco and alcohol abuse

Year	Number	Observed survival	Relative survival
*Total*			
6	652	94.3 (92.6–96.1)	96.8 (94.9–98.6)
7	562	89.5 (87.1–91.9)	94.1 (91.5–96.6)
8	466	85.2 (82.4–88.1)	92.1 (89.0–95.2)
9	406	81.3 (78.0–84.5)	90.3 (86.7–93.9)
10	344	76.1 (72.4–79.8)	87.1 (82.8–91.3)
*Adenocarcinoma*			
6	441	94.8 (92.7–96.9)	97.4 (95.2–99.5)
7	374	90.1 (88.0–93.5)	95.7 (92.7–98.6)
8	303	87.1 (83.8–90.5)	94.9 (91.2–98.5)
9	259	85.5 (81.9–89.0)	96.1 (92.0–100.0)
10	219	80.0 (75.7–84.3)	93.1 (88.1–98.2)
*Squamous cell carcinoma*			
6	206	93.2 (89.8–96.6)	95.3 (91.8–98.9)
7	183	86.6 (81.9–91.3)	90.5 (85.6–95.4)
8	160	81.2 (75.7–86.7)	86.5 (80.7–92.4)
9	144	73.3 (67.0–79.6)	79.7 (72.8–86.6)
10	122	68.5 (61.8–75.2)	76.0 (68.6–83.5)

## Discussion

This study found that esophageal cancer survivors have poorer survival than the corresponding background population, and the difference increased between 6 and 10 years after surgery. The reduction in relative survival seemed more pronounced in men than in women and in those with comorbidity than without, but was particularly marked among participants with a history of squamous cell carcinoma compared with adenocarcinoma of the esophagus.

This study benefits from a population-based design with the inclusion of all patients treated for esophageal cancer in Sweden who had survived for 5 years. This counteracts selection bias and facilitates generalizability. Another strength was the complete follow-up for survival, which rules out losses to follow-up. The well-maintained population registries in Sweden made it possible to compare the observed survival in the study cohort with that of the entire Swedish population of the corresponding age, sex, and calendar year. Among the limitations is the limited data available in the background population, which made it impossible to perform more targeted analyses and assess diseases that might contribute to the differences in survival. However, this was not the aim of the study. Although patients who have undergone surgery for esophageal cancer and survived for 5 years are considered cured of cancer, some late deaths due to tumor recurrences cannot be ruled out. However, any such deaths would occur earlier rather than later during the follow-up, and do not explain the main finding of a reduction in survival from 6 to 10 years after surgery. Finally, the statistical power did not allow robust analyses of survival more than 10 years after treatment.

This study yielded novel information to an area with gaps in knowledge. Previous studies investigating long-term survival in patients with esophageal cancer have examined survival up to 5 years after treatment, but to our knowledge, no study has compared the survival after 5 years with that of a corresponding general population.^19^

The results of the stratified analyses provide some clues that may help explain the findings. The most striking result was the substantial decline in relative survival in patients with a history of squamous cell carcinoma compared with those with adenocarcinoma. Heavy alcohol consumption and tobacco smoking, particularly in combination, are strong risk factors for squamous cell carcinoma, and these exposures are associated with several other diseases and generally poorer survival.^3,4^ This was supported by the finding of better survival in squamous cell carcinoma after exclusion of patients with patients with smoking and- alcohol-related diagnoses. Another factor that may contribute is the generally lower socioeconomic status of this patient category. Regarding adenocarcinoma, the main risk factor of gastro-esophageal reflux disease is not associated with decreased survival,^20,21^ while the risk factor of obesity reduces the overall survival to a limited degree, which could explain the slightly lower survival than the background population in this category.^22^ The relatively better survival in women compared with men may be at least partly explained by the knowledge that female patients with esophageal squamous cell carcinoma may be less likely to be heavy alcohol users than male patients.^23^ The lower decline in relative survival in recent calendar periods compared with earlier periods may be explained by a stricter selection of patients for surgery over time, who may be more fit and have fewer comorbidities compared with earlier years.^24,25^ These results indicate that survivors of esophageal cancer may benefit from recommendations of a healthier lifestyle, e.g., regarding tobacco smoking and alcohol consumption, and check-ups for potentially serious comorbidities. These recommendations may be especially relevant for men with a history of squamous cell carcinoma of the esophagus.

In conclusion, this Swedish nationwide and population-based cohort study shows that the life expectancy of esophageal cancer survivors is distinctly poorer than that of the corresponding general population, particularly in those with a history of squamous cell carcinoma of the esophagus and in men.

## Human and animal rights

This study is based on original data from medical records and health data registries. No previous study has compared the survival of esophageal cancer patients with that of a corresponding general population. The results indicate that survivors of esophageal cancer, especially men and those with a history of squamous cell carcinoma of the esophagus, benefit from recommendations of a healthier lifestyle, e.g., regarding tobacco smoking and alcohol consumption, and check-ups for potentially serious comorbidities.

## Data Availability

Data are available upon request after approval from the relevant authorities.
